# Case Report: Urachal inflammatory myofibroblastic tumor with bladder invasion

**DOI:** 10.3389/fonc.2026.1780322

**Published:** 2026-02-24

**Authors:** Xiaomeng Liu, Dongsheng Bai

**Affiliations:** Department of Urology, Capital Center for Children’s Health, Capital Medical University, Beijing, China

**Keywords:** anaplastic lymphoma kinase, bladder, invasion, inflammatory myofibroblastic tumor, surgery, urachus

## Abstract

**Background:**

Urachal inflammatory myofibroblastic tumor (IMT) is a rare mesenchymal neoplasm characterized by non-specific clinical and imaging features, which renders it susceptible to clinical misdiagnosis. Complete surgical resection is the preferred treatment method.

**Case presentation:**

We herein report a case of urachal IMT in a 12-year-old male child who presented with dysuria for 4 days and abnormal urine color for 2 days. Preoperative ultrasonography and contrast-enhanced computed tomography (CT) both suggested an infected urachal cyst. Laparoscopic resection of the urachal mass was initially performed; however, intraoperative exploration identified bladder invasion by the mass, prompting an additional partial cystectomy. Postoperative histopathological examination confirmed the diagnosis of urachal IMT with bladder invasion. No adjuvant therapy was administered postoperatively, and the patient remained free of recurrence and metastasis during a 6-month follow-up period.

**Conclusion:**

In the differential diagnosis of pediatric urachal lesions, IMT should be considered, especially when adjacent tissue invasion is present. Enhanced understanding of urachal IMT can assist clinicians in the early recognition and precise management of this tumor.

## 1 Introduction

Inflammatory myofibroblastic tumor (IMT) is a rare, low-grade malignant or borderline mesenchymal neoplasm characterized by the proliferation of fibroblastic and myofibroblastic spindle cells, accompanied by an inflammatory infiltrate composed of plasma cells, lymphocytes, eosinophils, and other inflammatory cells (1, 2). This tumor was first reported by Brunn et al. (3) in 1939. IMT was initially considered a non-neoplastic post-inflammatory lesion and confused with inflammatory pseudotumor (IPT) for an extended period (4). With the continuous accumulation of clinical and genetic evidence, particularly the discovery of anaplastic lymphoma kinase (ALK) gene rearrangements (5), this category of “IPT” was confirmed to be true neoplastic lesions with tumor properties. Currently, the World Health Organization (WHO) classification of soft tissue tumors expert group recommends unifying the nomenclature for these lesions as IMT (6).

Herein, we report a rare case of urachal IMT with bladder invasion, detailing its clinical, radiological, and histopathological profiles. This case reinforces that optimal management necessitates complete tumor resection and meticulous intraoperative exploration of the bladder.

## 2 Case report

**Figure 1:**
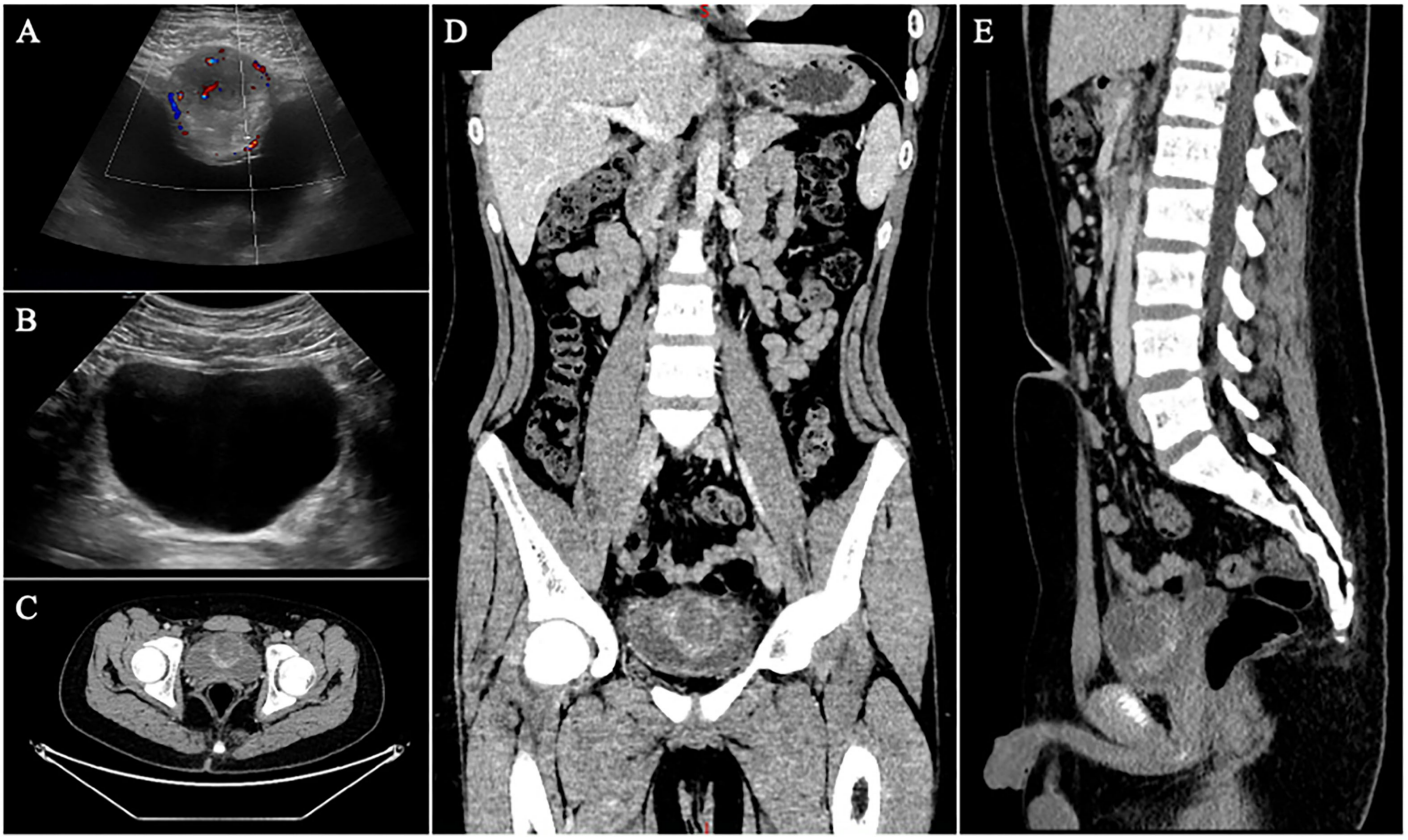
Imaging findings. **(A)** Preoperative ultrasound demonstrated a cystic lesion protruding into the bladder lumen from the anterior wall. The bladder mucosa is irregular, with increased echogenicity in the surrounding soft tissue and abundant blood flow signals in the superior wall. **(B)** At 6-month postoperative follow-up, ultrasound showed focal blurring of the anterior bladder wall with no evidence of abnormal echoic lesions. **(C–E)**. Preoperative contrast-enhanced CT showed an ovoid mass in the anterosuperior bladder wall with heterogeneous peripheral rim enhancement.

A 12-year-old male was admitted to our hospital with a 4-day history of dysuria and a 2-day history of abnormal urine color. The patient experienced dysuria without obvious precipitating factors four days prior to admission, with no fever, abdominal pain, urinary frequency, or urgency. Two days before admission, he developed gross hematuria throughout the entire stream, bright red in color with visible blood clots. The patient presented to the urology clinic of our hospital for consultation. Routine blood test results revealed a white blood cell count of 5.94×10^9^/L, a neutrophil percentage of 59%, and a C-reactive protein (CRP) level of 2.32 mg/L. Urinalysis revealed diffuse red blood cells on microscopic examination with uniform morphological features, and white blood cells were 0 per high-power field. Abdominal ultrasound demonstrated a 4.3×4.2×4.2 cm cystic lesion arising from the anterior wall of the bladder, protruding into the bladder lumen with an irregular mucosal layer and enhanced echogenicity of the surrounding soft tissue. A strip-like hypoechoic area extended laterally toward the umbilicus. Color Doppler flow imaging revealed relatively abundant blood flow signals in the superior wall of the cystic lesion. The ultrasound impression was a urachal cyst with infection (**Figures 1A**). The patient was subsequently admitted with a provisional diagnosis of *urachal cyst*. Physical examination on admission was unremarkable. Post-admission contrast-enhanced CT scan showed an elliptical lesion on the anterosuperior wall of the bladder, measuring 3.4×4.1×5.1 cm. The borders were ill-defined, with central cystic components showing a density of 15 HU on unenhanced CT and 33 HU on contrast-enhanced CT, with the lesion showing heterogeneous, peripheral and mild enhancement. The anterior bladder wall was indented and compressed. The CT impression was an extrinsic lesion on the anterosuperior wall of the bladder, predominantly cystic, possibly a urachal cyst with infection (**Figures 1C–E**).

**Figure 2:**
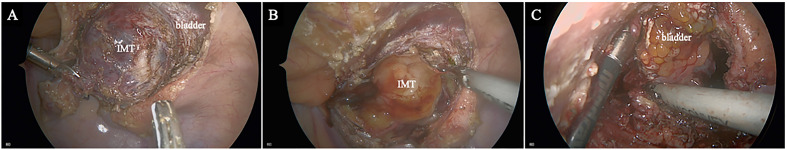
Intraoperative laparoscopic view. **(A)** Gross examination revealed tumor invasion into the muscularis propria of the bladder. **(B)** The tumor was filled with brownish-yellow, mucoid, gelatinous contents. **(C)** Incision of the bladder revealed numerous polypoid lesions on the mucosal layer.

Laparoscopic resection of the urachal mass was performed under general anesthesia. Intraoperatively, a roundish mass measuring approximately 4×4×5 cm was observed at the distal end of the urachus, anterosuperior to the bladder. During tumor dissection, the bladder invasion was confirmed, with internal contents appearing brownish-yellow, mucoid, and gelatinous (**Figures 2A, B**); accordingly, a portion of the tumor tissue was resected for frozen section pathological examination. The frozen section report indicated that the morphological features were consistent with spindle cell proliferative lesions, accompanied by myxoid degeneration and diffuse inflammatory cell infiltration; mild to moderate cellular pleomorphism was noted, and the lesions extended to the surrounding muscular tissue. Given these findings, we suspected the presence of malignant components within the tumor. Combined with preoperative ultrasound demonstrating an irregular bladder mucosal layer, cystotomy exploration was performed. Intraoperatively, numerous vegetations were identified in the bladder mucosal layer following cystotomy (**Figure 2C**). Ultimately, complete resection of the urachal IMT and the partially invaded bladder tissue was successfully accomplished.

**Figure 3:**
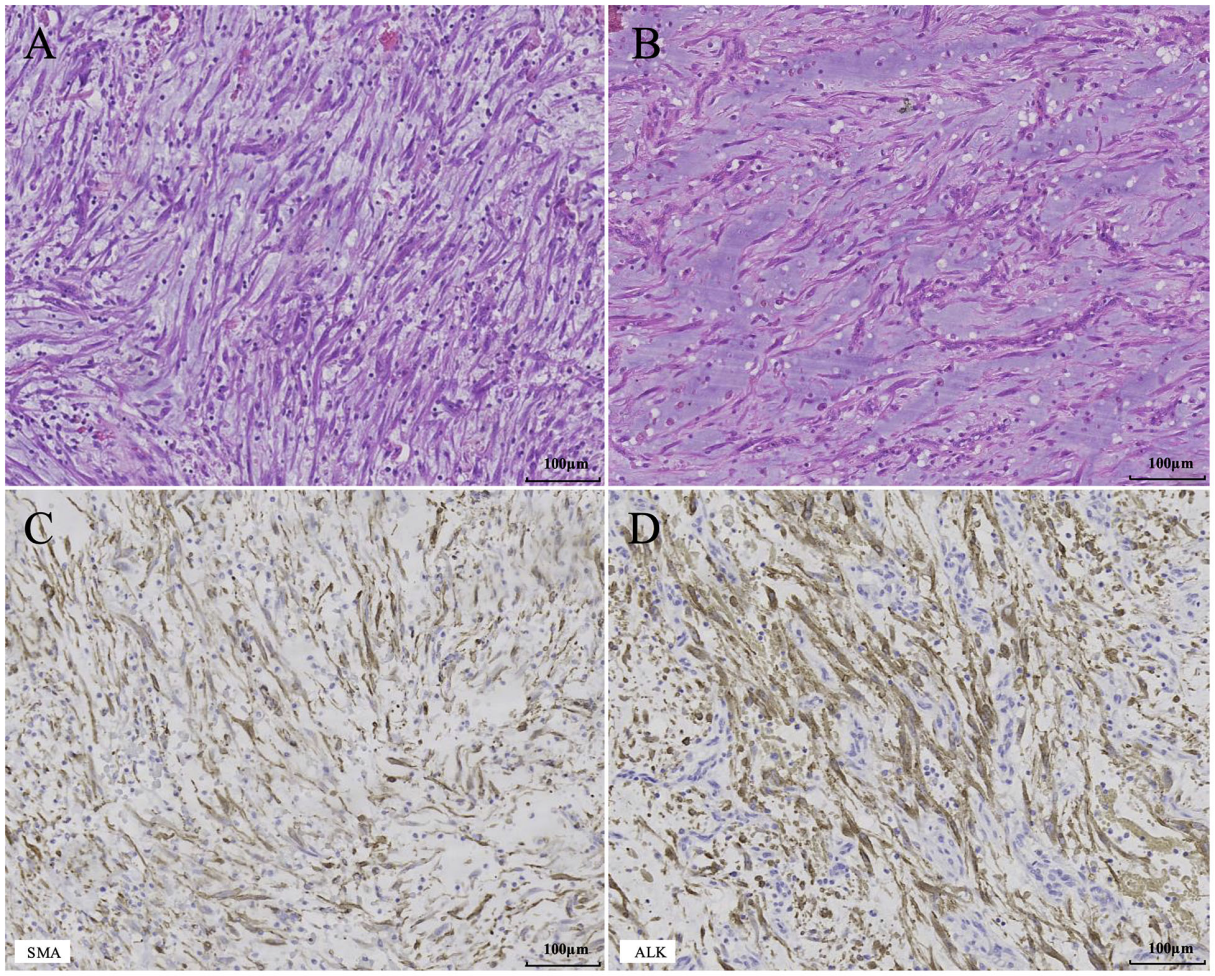
Histopathological findings of the tumor. **(A)** Representative image shows numerous spindle cells arranged in fascicles or storiform patterns, with densely distributed inflammatory cells between the spindle cells (H&E stain, ×200). **(B)** Representative image reveals spindle cells loosely arranged in myxoid stroma with inflammatory cell infiltration (H&E stain, ×200). **(C, D)**. Representative images show strong positive expression of SMA and ALK (IHC stain, ×200).

Postoperative histopathological findings were compatible with a spindle cell proliferative lesion, accompanied by extensive myxoid degeneration and diffuse inflammatory cell infiltration. The spindle cells exhibited atypia, with the lesion invading the bladder mucosa and muscular layer. Combined with the immunohistochemistry (IHC) results: smooth muscle actin (SMA) (+), ALK (+), Ki-67 antigen (index 40%), vimentin (+), CD34 (-), desmin (-), pan-tyrosine receptor kinase (-), S100 calcium-binding protein (-), myogenic differentiation 1 protein (-), the diagnosis was consistent with IMT (**Figure 3**). The patient had an uneventful postoperative recovery on postoperative day 7. Following urethral catheter removal, the patient achieved unobstructed micturition without postoperative complications related to partial cystectomy, including urinary frequency and urgency, and was discharged successfully. At the 6-month postoperative follow-up, a repeat abdominal ultrasound demonstrated no evidence of tumor recurrence (**Figure 1B**), and no pulmonary metastasis was observed on chest CT scan.

## 3 Discussion

IMT occurs predominantly in children and adolescents, most frequently in the lung, and may also arise in other anatomic sites, though it remains a rare tumor overall (2, 7). Within the genitourinary system, the most common site for IMT is the bladder, while urachal IMT is extremely rare (8). The clinical and radiological manifestations of IMT lack specificity and vary depending on the site of origin (2). Urachal IMT may clinically present as an abdominal mass, abdominal pain, hematuria, dysuria, etc. In terms of imaging findings such as CT or magnetic resonance imaging, a cystic-solid mass can be observed in the anterosuperior region of the bladder, with heterogeneous peripheral enhancement on contrast-enhanced scans. This appearance is easily misdiagnosed as urachal cyst, urachal carcinoma, or bladder cancer (9–14). Pathological examination remains the gold standard for diagnosing IMT (4). Microscopically, IMT exhibits three typical histological patterns (15): (1) Myxoid/vascular pattern: Spindle cells are loosely arranged in a myxoid stroma rich in blood vessels, accompanied by inflammatory cell infiltration; (2) Cellular spindle cell pattern: A large number of spindle cells are arranged in fascicles or storiform patterns, with inflammatory cells densely distributed among the spindle cells; (3) Hypocellular fibrous pattern: Scattered spindle cells are seen within a dense collagenous stroma with sparse inflammatory cells. These patterns may occur alone or coexist in combination. Immunohistochemically, IMT exhibits characteristics of myofibroblastic differentiation. In the majority of cases, positive immunohistochemical expression of SMA, MSA, Vimentin, and Desmin can be observed. ALK protein immunoreactivity is detectable in approximately 36-73% of cases, often showing strong positivity, and its expression pattern correlates with its fusion partner. (2). Genetically, IMT involves various gene rearrangements, with ALK gene rearrangements found in about 50-70% of cases (2). The ALK gene, located on chromosome 2p23, undergoes chromosomal translocation and fuses with various partner genes. These gene fusions lead to activation and overexpression of the ALK protein’s tyrosine kinase domain, thereby driving tumorigenesis (1). In this case, preoperative imaging identified a mass located on the anterosuperior wall of the urinary bladder, and contrast-enhanced CT demonstrated heterogeneous ring enhancement of the lesion. Intraoperative exploration confirmed bladder wall invasion, and postoperative histopathological examination revealed spindle cell proliferation accompanied by inflammatory cell infiltration, which exhibited the typical morphological features of the cellular spindle cell pattern and myxoid/vascular pattern, consistent with IMT. IHC staining confirmed SMA (+), ALK (+), and Vimentin (+), thus consistent with the diagnosis of urachal IMT with bladder invasion.

Radical surgical resection is the primary treatment for IMT (1, 2). Urachal IMT often requires partial cystectomy due to bladder invasion or close adhesion to the bladder (9–14). Therefore, we recommend routine cystoscopy for patients with a preoperative suspicion of urachal IMT, as it aids in evaluating bladder involvement and guiding the selection of the surgical approach. If tumor invasion of the bladder is identified, a preoperative tissue biopsy can be performed to confirm the diagnosis, or the feasibility of partial cystectomy can be assessed based on the extent and location of bladder involvement. Radiotherapy and corticosteroid therapy can serve as adjuvant treatments for patients with incomplete resection or those who are not surgical candidates (2). For patients with advanced disease requiring systemic therapy, chemotherapy represents an effective treatment option (16, 17). The emergence of ALK inhibitors represents a significant breakthrough in IMT treatment. Current guidelines recommend ALK inhibitors as first-line therapy for patients with ALK-rearranged, advanced, recurrent, metastatic, or inoperable IMT (18). For ALK-positive patients with urachal IMT who have extensive bladder invasion, or invasion of the trigone and bladder neck, and for whom partial cystectomy would result in significant impairment of bladder capacity or micturition function, tumor debulking combined with ALK inhibitor therapy may represent an effective therapeutic option, albeit one that requires further clinical evaluation. Most patients who undergo complete tumor resection have a favorable prognosis, with an overall 5-year survival rate exceeding 95% (16, 19). Distant metastasis in IMT is less than 5%, with the lung and brain being the most common sites (2). Studies suggest that ALK protein expression is associated with dis-tant metastasis in IMT, ALK-negative patients have a higher rate of distant metastasis, but this expression is not related to local recurrence (20). The recurrence of IMT is primarily associated with the tumor location and the feasibility of complete resection (21). Preoperatively, the patient was diagnosed with an infected urachal cyst; however, intraoperative exploration revealed the lesion to be a cystic-solid mass with brownish-yellow, mucoid, and gelatinous contents-findings inconsistent with the typical manifestations of an infected urachal cyst, which presents as a purely cystic structure filled with purulent or pyopurulent fluid. Additionally, the lesion was found to have invaded the urinary bladder. In such circumstances, frozen section pathological examination, as performed intraoperatively in this case, is warranted, as it can preliminarily determine the tumor nature and the extent of adjacent tissue involvement, thereby guiding subsequent surgical decision-making. Ultimately, complete resection of the urachal IMT and the focally invaded portion of the urinary bladder was achieved. No adjuvant therapy was administered postoperatively.

This patient demonstrated a high Ki-67 proliferation index (40%) and bladder wall invasion, necessitating a differential diagnosis with epithelioid inflammatory myofibroblastic sarcoma (EIMS). EIMS is an aggressive variant of IMT, histologically characterized by epithelioid cells with vesicular nuclei and prominent nucleoli within a prominent myxoid stroma, with focal necrosis observed in approximately 50% of cases (1). These features were inconsistent with the histological findings in the present case. Moreover, Ki-67 expression has been correlated with the prognosis of IMT (22). This patient remained free of tumor recurrence or metastasis throughout the 6-month postoperative follow-up period.

This case report documents a rare malignant tumor originating from the urachus. By reporting this case in the literature, it supplements the clinical data related to urachal IMT, enhances clinicians’ understanding of this tumor, and provides reference for its diagnosis and management. However, as IMT encompasses multiple gene fusion subtypes, the IHC findings in this case may not be representative of all instances, which constitutes the primary limitation of this report.

Taken together, urachal IMT is exceedingly rare and presents with non-specific clinical and imaging manifestations, making it susceptible to being misdiagnosed as a urachal cyst or bladder cancer, which can result in either delayed or unnecessarily aggressive treatment. For pediatric patients with or without hematuria and a mass in the anterosuperior wall of the bladder showing heterogeneous peripheral enhancement on contrast-enhanced CT, the possibility of urachal IMT should be considered. Definitive diagnosis requires histopathological examination. Although IHC can help detect ALK protein expression, a negative IHC result does not exclude IMT. For ALK-negative patients, further molecular biological testing may be required to confirm the diagnosis. Complete surgical resection is the primary therapeutic approach for urachal IMT. Since some IMTs display invasive characteristics, intraoperative cystoscopy is recommended to evaluate for bladder involvement. Given the propensity of IMT for recurrence, long-term follow-up surveillance is necessary for patients.

## 4 Conclusion

Urachal IMT lacks specific clinical and imaging manifestations, and its definitive diagnosis relies on pathological examination. Complete surgical resection of the tumor is the first-line treatment modality. Given the high propensity of this tumor for bladder invasion, intraoperative cystoscopy is considered an essential procedural step.

## Data Availability

The original contributions presented in the study are included in the article/supplementary material. Further inquiries can be directed to the corresponding author/s.
